# Comparison of Four Validated Nomograms (Memorial Sloan Kettering Cancer Center, Briganti 2012, 2017, and 2019) Predicting Lymph Node Invasion in Patients with High-Risk Prostate Cancer Candidates for Radical Prostatectomy and Extended Pelvic Lymph Node Dissection: Clinical Experience and Review of the Literature

**DOI:** 10.3390/cancers15061683

**Published:** 2023-03-09

**Authors:** Giovanni Battista Di Pierro, Stefano Salciccia, Marco Frisenda, Antonio Tufano, Alessandro Sciarra, Emiliano Scarrone, Francesco Del Giudice, Vincenzo Asero, Giulio Bevilacqua, Martina Moriconi, Antonio Carbone, Antonio Pastore, Stefano Signore, Pierluigi Bove, Flavio Forte, Paolo Emiliozzi, Andrea Tubaro, Cosimo De Nunzio, Vittorio Canale

**Affiliations:** 1 Department ‘’Materno Infantile e Scienze Urologiche’’, Policlinico Umberto I, Sapienza University of Rome, 00161 Rome, Italy; 2 Department of Urology, ICOT Latina, University Sapienza, 04100 Latina, Italy; 3 Sant’Eugenio Hospital, 00144 Rome, Italy; 4 San Carlo di Nancy Hospital, 00165 Rome, Italy; 5 Vannini Hospital, 00177 Rome, Italy; 6 San Camillo Hospital, 00152 Rome, Italy; 7 Department of Urology, Ospedale Sant’Andrea, University Sapienza, 00189 Rome, Italy

**Keywords:** prostatic neoplasm, extended pelvic lymph node dissection, nomograms, radical prostatectomy

## Abstract

**Simple Summary:**

The indication for ePLND at the time of RP is based on a risk assessment of LNI through validated nomograms such as the MSKCC; Briganti 2012; Briganti 2017 and Briganti 2019. However, in daily practice, a relevant percentage of cases, including those with the high-risk disease, show no LNI at the final histopathological assay pathology (pN0) after ePLND. Furthermore, currently available evidence does not demonstrate the superiority of one nomogram over the others, and there is still lacking data to support the routine use of one predictive model over another, even in more aggressive diseases. Therefore, we evaluated the accuracy of the most used nomograms (MSKCC, Briganti 2012, Briganti 2017, and Briganti 2019) for predicting LNI and compared them in our sub-cohort of high-risk PC patients treated with ePLND. We found that the predictive performance of the four nomograms as well as their ability to avoid unnecessary ePLND, are virtually the same, even in high-risk PC patients.

**Abstract:**

Background: The indication for extended pelvic lymph node dissection (ePLND) at the time of radical prostatectomy (RP) is based on nomograms predicting the risk of lymph node invasion (LNI). However, limited data are available on the comparison of these predictive models in high-risk prostate cancer (PC) patients. Therefore, we compared the accuracy of the most used nomograms (MSKCC, Briganti 2012, 2017, and 2019) in the setting of high-risk PC patients submitted to ePLND. Methods: 150 patients with high-risk PC disease treated from 2019 to 2022 were included. Before RP + ePLND, we assessed the MSKCC, Briganti 2012, 2017, and 2019 nomograms for each patient, and we compared the prediction of LNI with the final histopathological analysis of the ePLND using pathologic results as a reference. Results: LNI was found in 39 patients (26%), and 71.3% were cT2. The percentage of patients with estimated LNI risk above the cut-off was significantly higher in pN+ cases than in pN0 for all Briganti nomograms. The percentage of patients at risk of LNI, according to Briganti Nomogram (2012, 2017, and 2019), was significantly higher in pN+ cases than in pN0 (*p* < 0.04), while MSKCC prediction didn’t vary significantly between pN0 and pN+ groups (*p* = 0.2). All nomograms showed high sensitivity (Se > 0.90), low specificity (Sp < 0.20), and similar AUC (range: 0.526–0.573) in predicting pN+. Particularly, 74% of cases patients with MSKCC estimated risk > 7% showed pN0 compared to 71% with Briganti 2012 > 5%, 69% with Briganti 2017 > 7%, and 70% with Briganti 2019 > 7%. Conclusions: Despite the high-risk disease, in our patients treated with ePLND emerges a still high number of pN0 cases and a similar low specificity of nomograms in predicting LNI.

## 1. Introduction

Prostate cancer (PC) is the most common urological cancer in men. In patients with non-metastatic disease eligible for radical prostatectomy (RP), current European Urological Association (EAU) guidelines recommend performing an extended pelvic lymph node dissection (ePLND) according to the risk for lymph node invasion (LNI) estimated by validated nomograms [1,2,3,4]. Overall, although current evidence shows no improved survival when associating an ePLND to RP [5] and no significant differences in terms of oncological outcomes between limited PLND and ePLND [6], the prognostic and staging role of ePLND is undiscussed [2,6]. Indeed, despite recent efforts to devlop new imaging techniques for nodal staging, they do not represent reliable tool to predict LNI due to (the) lack of sensitivity, and ePLND still remains the gold standard for the detection of lymph node invasion [2,5,6,7,8,9,10].

Since the performance of ePLND is associated with increased morbidity and higher costs [5], in everyday clinical practice, the indication for ePLND is usually assessed by available nomograms such as the Briganti 2012, Briganti 2017, Briganti 2019 or the MSKCC nomograms [2,3,4,7,8]. Recently, the Briganti 2019 nomogram has also incorporated the multiparametric magnetic resonance imaging (mpMRI) findings and mpMRI-targeted biopsy as parameters to be considered, with a threshold of 7% which would result in missing 1.5% of patients with LNI [7].

To date, in the literature, there are several studies investigating the accuracy of nomograms [8,11,12,13,14,15,16,17,18,19,20,21]. However, few studies have directly compared the accuracy of MSKCC, Briganti 2012, 2017, and 2019 nomograms. In addition, limited data are available on the comparison of these predictive models in a selected population with only high-risk prostate cancer (PC).

The aim of the present study is to compare the accuracy of the most used nomograms (MSKCC, Briganti 2012, 2017, and 2019) predicting LNI, specifically in patients with high-risk PC candidates to ePLND at the time of RP.

## 2. Materials and Methods

From 2019 to 2022, all patients with high-risk PC treated with RP and ePLND at our departments were retrospectively examined and included in the analysis. High-risk PC was defined according to both the EAU and D’Amico classification. All diagnostic and therapeutic procedures reflected our routine clinical practice in a department at a high volume for the management of PC disease. Inclusion criteria were: diagnosis of high-risk PC; no distant metastases at clinical staging; RP as chosen primary treatment decision after discussion of treatment options. Exclusion criteria were previous or current androgen deprivation therapy, chemotherapies, pelvic radiation therapy, or treatments with other agents that could influence prostate tumor growth and diffusion. 

### 2.1. Nomogram Evaluation

Briganti 2012 [3], Briganti 2017 [4], Briganti 2019 [7], and MSKCC [8] nomograms were evaluated using preoperative clinical and pathological features in order to establish the probability of LNI. As previously described, the MSKCC nomogram is based on preoperative PSA, clinical stage, primary and secondary biopsy Gleason pattern as well as negative and positive biopsy cores; the Briganti 2012 nomogram is based on pretreatment preoperative PSA, clinical stage, primary and secondary biopsy Gleason score and percentage of positive cores; the Briganti 2017 based on includes preoperative PSA; clinical stage, biopsy Gleason grade, percentage of positive cores with the highest and with the lowest grade disease; the Briganti 2019 is based on pretreatment preoperative PSA, clinical stage, grade group at MR-targeted biopsy, the maximum diameter of the index lesion at mpMR, and the percentage of cores with clinically significant PC at systematic biopsy.

### 2.2. Surgical Procedure and Pathologic Evaluation

All the Procedures were performed using a standard robot-assisted (RARP) or pure laparoscopic (LRP) radical prostatectomy approach. Anatomical ePLND was performed in a standardized manner as previously described and included the removal of the nodes overlying the external iliac artery and vein, the nodes within the obturator fossa, and the nodes medial and lateral nodes to the internal iliac artery [22].

All histological specimens of prostatic biopsy and RP were analyzed by our uro-pathologists, with a long experience in the PC field. In all cases, they reported the Gleason score and grade groups according to the World Health Organization (WHO)/ISUP guidelines at biopsy and at surgery, pathologic staging using TNM classification, surgical margin (SM) status, and perineural invasion (PNI) were routinely defined in all cases. Lymph node involvement was defined as the presence of positive pelvic lymph nodes for PC at the histopathological assay. The outcome of our study was lymph node involvement, defined as the presence of positive pelvic lymph nodes for PC at final pathology. The number of lymph nodes removed at surgery and the percentage of positive LNs for PC in pN+ cases were reported. 

### 2.3. Statistical Analysis

Data analyses were performed using STATA version 17.0 (Stata Corp., College Station, TX, USA).

Descriptive statistical methods such as number and percentage of cases, mean ± SD, median, and range were used. For the comparison of quantitative data, a Mann–Whitney test was used, whereas for qualitative data, a Fisher’s Exact test and chi-square test were used. Pearson correlation analysis was also performed. We assessed the accuracy of the available nomograms MSKCC, Briganti 2012, Briganti 2017, and Briganti 2019 to predict LNI defined at final pathology. Regression coefficients were used to calculate the risk of LN positivity according to each model, and the discrimination accuracy of these models was quantified using the area under the receiver operating characteristic (ROC) curve (AUC). Sensitivity, specificity, positive predictive value (PPV), and negative predictive value (NPV) of the different clinical variables in predicting pathologic LN status were evaluated. Statistical significance was fixed at *p* < 0.05.

## 3. Results

Overall, 150 patients with high-risk PC submitted to RP with ePLND were included in the present analysis. The baseline characteristics of the included population are described in Table 1. PIRADS 4 was the most frequently observed (47.5%) at mpRMI pattern; 84% of them were cN0, and 71.3% were cT2.

At final pathology, LNI was found in 39 patients (26.1%), and the mean ± SD percentage of positive LNs was 16.1 ± 12.9 (Table 1). 

The percentage of patients with an estimated risk for N+ at nomograms above the recommended cut-off threshold was significantly higher in pN+ cases than in pN0 for all Briganti nomograms (*p* < 0.04) but not for MSKCC nomogram (*p* = 0.2). The site of positive LNs at final pathology analysis was not described in most cases and was simply classified as left or right.

When considering nomograms results as continuous variables, mean ± SD estimated risk for pN+ showed some differences among MSKCC (33.5 ± 19.7), Briganti 2012 (26.1 ± 19.7), Briganti 2017 (43.3 ± 25.4) and Briganti 2019 (24.9 ± 20.0) nomograms (Table 1). 

### 3.1. Comparative Analysis between pN0 and pN+ Cases

A comparative analysis between pN0 and pN+ cases is reported in Table 2.

The mean ± SD number of LNs removed was similar between pN0 (23.8 ± 8.4) and pN+ (25.0 ± 8.1) cases (*p* = 0.2). Only preoperative PSA and the maximal percentage of PC tissue per core at biopsy were significantly higher in pN+ (mean value 17.6 ± 15.1 ng/mL and 71.3 ± 24.1, respectively) when compared to pN0 cases (mean value 10.9 ± 10.0 ng/mL and 56.5 ± 27.6, respectively) cases (all *p* < 0.01). 

Considering nomograms results as a continuous variable, none of the four preoperative nomograms (although mean values were always higher in pN+ than in the pN0 group) showed percentages of estimated risk for pN+ significantly different between pN0 and pN+ cases (all *p* > 0.05) (Table 2). 

Pearson correlation analysis showed no statistically significant correlation between the pN result and each of the four nomograms examined as continuous variables (*p* > 0.1), whereas a statistically significant correlation was found with preoperative PSA (r = 0.2155; *p* = 0.008). 

Different pathologic parameters at RP, such as pT stage, ISUP grading, and surgical margins but not the number of nodes surgically removed (r = 0.0793, *p* = 0.334), significantly correlated with pN status (Table 3).

### 3.2. Sensitivity, Specificity, PPV, NPV, and AUC Results in Predicting pN Status

The performance of the four nomograms in predicting pN status at final pathology is reported in Table 4. 

**Figure 1:**
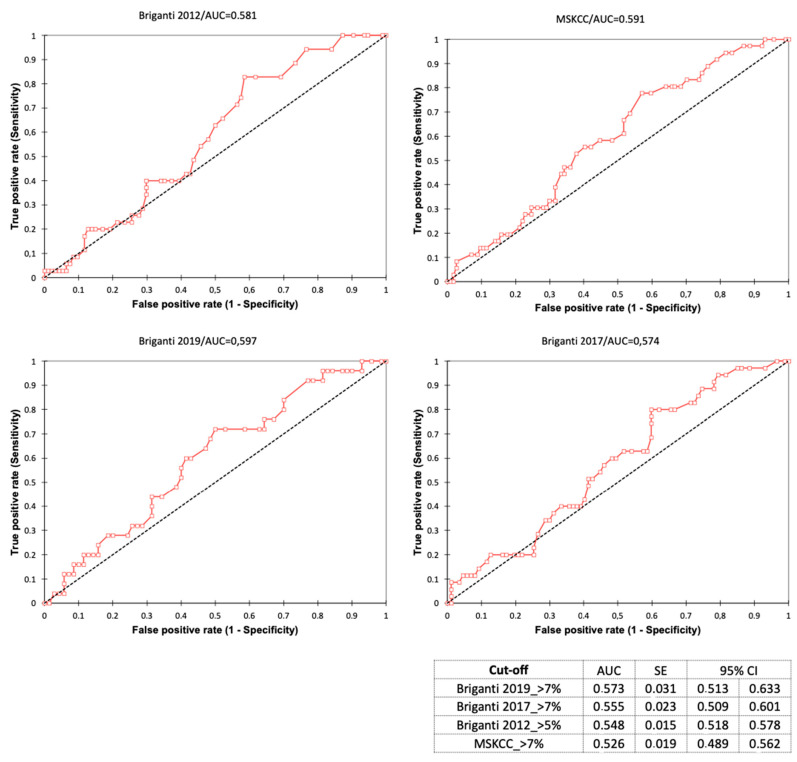
Receiver operating characteristic (ROC) curve and relative Area Under the Curve (AUC) in predicting LNI (pN+) of the currently available MSKCC, Briganti 2012, Briganti 2017, and Briganti 2019 nomograms.

In our population, nomograms showed similar high sensitivity (0.973, 0.991, 0.973, and 0.959, respectively, for MSKCC, Briganti 2012, Briganti 2017, and Briganti 2019) and low specificity (0.078, 0.093, 0.140 and 0.183 respectively for MSKCC, Briganti 2012, Briganti 2017 and Briganti 2019) at the recommended threshold of estimated risk (of >5% or >7%). AUC values were similar, with 0.526, 0.548, 0.555, and 0.573 for the four nomograms, respectively (Figure 1).

**Figure 2:**
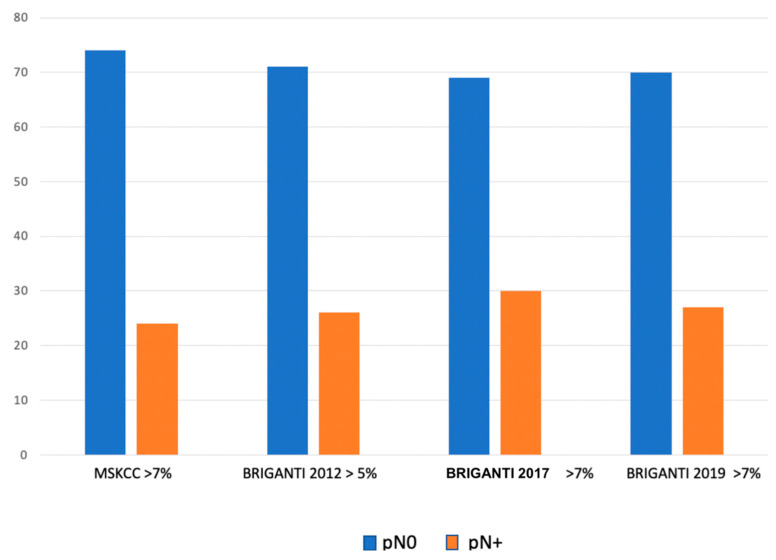
Bar-chart showing pN0 and pN+ cases distribution according to the MSKCC (at estimated risk > 7%), Briganti 2012 (at estimated risk > 5%), Briganti 2017 (at the estimated risk > 7%), Briganti 2019 (at estimated risk > 7%) nomograms.

74% of cases with MSKCC estimated risk > 7% showed no LNI (pN0) at final pathology compared to 71% of cases with Briganti 2012 > 5%, 69% with Briganti 2017 > 7% and, 70% with Briganti 2019 > 7% (Figure 2). When the MSKCC nomogram was performed, 74% of patients with more than 7% risk of LNI showed no LNI at histopathological node assay.

### 3.3. Regression Analysis: Predictors for pN+ Result at Final Pathology

A logistic regression analysis was carried out to identify predictors of positive PLN involvement at final pathology (pN+) (Table 5).

At the univariable analysis, the risk of pN+ significantly increased according to the pT stage, 6.7 times in pT3a (*p* = 0.003) and 14.1 times in pT3b cases (*p* = 0.001).

According to the various nomograms, the risk of pN+ increased 3.0 times for an MSKCC estimated risk > 7%, 7.7 times for a Briganti 2012 estimated risk > 5%, 5.6 times for a Briganti 2017 estimated risk > 7% and 5.5 times for a Briganti 2019 estimated risk > 7%, without statical significance (all *p* > 0.1).

At the multivariable analysis, the pT stage maintained an independent predictive value in terms of risk for pN+ (*p* < 0.05). 

## 4. Discussion

According to EAU guidelines [2], the indication for ePLND at the time of RP is based on risk assessment by validated nomograms such as the MSKCC, Briganti 2012, Briganti 2017, and Briganti 2019 [3,4,7,8]. Using a cut-off of 5% or 7% in terms of estimated risk for pN+ results in missing a very low percentage of cases with LNI [3,4,7,8]. On the other hand, in daily practice, a relevant percentage of cases show no LNI at the final histopathological assay (pN0) after ePLND [2,3,4,5,6,7,8,9,10,11,12,13,14,15,16,17,18,19,20,21]. In fact, it has been demonstrated that when the choice of whether to perform ePLND relies on well-established preoperative nomograms, most patients, including those with high-risk diseases, have no LNI at final pathology [2,3,4,5,6,7,8,9,10,11,12,13,14,15,16,17,18,19,20,21]. 

To date, various comparisons among nomograms in different patient cohorts have been published (Table 6). 

M. Bandini et al. [12] compared four different nomograms: Cagiannos, Godoy, the 2012 Briganti, and the online MSKCC nomograms. Despite several comprehensive analytical steps, they did not prove the superiority of one nomogram over another. Furthermore, all nomograms achieved the same accuracy for predicting LNI, and their ability to avoid unnecessary PLND was similar. 

Hueting et al. [13] performed an external validation of 16 predictive models in 1001 Dutch patients with PCa, excluding the Briganti 2017 and 2019 nomograms. LNI was identified in 276 patients (28%). They showed that the Briganti 2012 (AUC 0.76) and MSKCC nomograms (AUC 0.75) were the most accurate, with similar miscalibration with a tendency to underestimation. No direct comparison between nomograms, however, was performed. 

Again, Oderda et al. [15] performed a multi-institutional external validation of several nomograms for the prediction of LNI. Overall, 1158 patients (9.6%) had LNI, with a mean of 17.7 and 3.2 resected and positive nodes, respectively. No significant differences in AUCs were observed between the MSKCC (0.83), Briganti 2012 (0.83), Partin 2016 (0.78), Yale (0.80), Briganti 2017 (0.80), and Briganti 2019 (0.76) models.

However, these results do not match with Hinev et al. [11] findings which surprisingly showed that the 2012 Briganti nomogram is far superior to the MSKCC nomogram, reporting a calculated AUC of 0.875 vs. 0.77. More recently, Diamand et al. [14] found the same AUC of 0.8 for both Briganti 2012 and 2019 but with a better net benefit for the 2019 model.

Similarly, in a recent external validation of the Briganti 2019 nomogram, this tool was characterized by higher AUC compared to the Briganti 2012 and 2017 nomograms and the Memorial Sloan Kettering Cancer Center risk calculator (79% vs. 75% vs. 65% vs. 74%) and demonstrated the highest net benefit on decision curve analyses [18]. 

The results of this study [18] suggest that adding valid imaging, such as mpMRI, can improve the predictive power of these tools. 

However, available evidence does not demonstrate the superiority of one nomogram over the others. No data exist to support the routine use of one predictive model over another, even in more aggressive diseases.

Based on these considerations, we aimed to evaluate the accuracy of the most used nomograms for predicting LNI and to compare them in our sub-cohort of high-risk PC patients treated with ePLND. 

Overall, we found that the predictive performance of the MSKCC, Briganti 2012, 2017, and 2019 nomograms about LNI are virtually the same. Indeed, despite higher PCa aggressiveness in the present cohort, our findings are in line with those in other series reporting on the general population: the AUC values were similar (0.526, 0.548, 0.555, and 0.573 for the MSKCC, Briganti 2012, Briganti 2017, and Briganti 2019 models, respectively) and the four nomograms showed similar high sensitivity (0.973, 0.991, 0.973, and 0.959 for MSKCC, Briganti 2012, Briganti 2017, and Briganti 2019, respectively) and very low specificity (0.078, 0.093, 0.140, and 0.183 for MSKCC, Briganti 2012, Briganti 2017, and Briganti 2019, respectively).

Theoretically, in the present sub-cohort of patients, the role of ePLND should be undiscussed, and, therefore, preoperative nomograms should play a limited role in deciding whether to perform ePLND. Specifically, when analyzing patients’ characteristics, we observed that most of them showed unfavorable tumor features: 74% of cases showed >pT2 disease and high ISUP grade (59% of patients with grade > 3) at final pathology. Nonetheless, it is relevant to underline that 74% of cases with MSKCC estimated risk > 7% showed no LNI (pN0) at final pathology compared to 71% of cases with Briganti 2012 > 5%, 69% with Briganti 2017 > 7% and 70% with Briganti 2019 > 7%. These findings demonstrate that these models tend to overestimate the LNI risk, also in patients diagnosed with high-risk disease. In other words, their ability to avoid unnecessary ePLND is similar; also, in this surgical setting, where we should expect greater accuracy. In addition, our results further corroborate that mpMRI may not be capable of detecting small pelvic lymph node metastases and MRI data on the index lesion are not enough for accurate LNI prediction: mpMRI is highly operator-dependent, and its misinterpretation could account for the performance of the Briganti 2019 nomogram as compared to older nomograms [23]. Therefore, this study confirms that, in a real-life setting, mpMRI and MRI-targeted biopsy may provide a limited additional value in improving the accuracy of clinical predictors of LNI. Indeed, it is well-established that the sensitivity and specificity of mpMRI in the direct detection of metastatic nodes, based on morphological characteristics, are not sufficient [24]. Moreover, in a previous experience, we observed that even a defined standardization of lymph node involvement, such as node rads, did not show superior results to the current nomograms, clearly suggesting the limits of MRI in the lymph node staging (25). Therefore, the question of whether we really need to perform so many ePLNDs remains unanswered. The answer will probably come from future refinements and the widespread adoption of PSMA-PET, which has proven to be superior to conventional imaging for high-risk PCa patients with pelvic nodal metastases [25,26,27,28,29]. It is likely that, in the future, ePLND will be guided directly by PSMA-PET or nomograms integrating PSMA PET/CT data. In fact, as recently demonstrated by Meijer et al., the addition of PSMA-PET to the previously developed nomograms showed substantially improved predictive performances, which suggests that PSMA-PET is a likely future candidate for a modern predictive nomogram [16]. Moreover, in a recent comparative study between ^68^ Ga-PSMA PET/CT and mpMRI in the diagnosis of lymph node metastases, Franklin A et al. observed that preoperative ^68^ Ga-PSMA/PET CT was more sensitive in identifying histological pelvic LNM than 3-T mpMRI. Moreover, they observed that men with a negative ^68^ Ga-PSMA PET/CT have a lower risk of LNI than predicted with MSKCC and Briganti nomograms [27].

Our study is not devoid of limitations: (I) this is a retrospective analysis; (II) some data, such as localization of positive LNs, were not available; (III) multiple surgeons performed ePLND, multiple pathologists reported on the histopathology in the RP and ePLND specimens, and scans were reported by multiple radiologists. Nonetheless, all patients were diagnosed and treated at high-volume tertiary referral centers by experienced surgeons and dedicated expert radiologists and uro-pathologists.

## 5. Conclusions

Although we considered only high-risk PC cases candidate for ePLND, a high percentage of them continues to show no LNI at final histopathology. In addition, we confirm the similar predictive value in terms of LNI estimation among the four most frequently used validated nomograms, with similar high sensitivity but low specificity. 

## Figures and Tables

**Table 1:** Baseline characteristics of the whole population.

Number of cases	150
Age (years)	64.7 ± 5.35; 66: (49–71)
BMI	25.4 ± 3.3; 26: (20.9–37.8)
Preoperative total PSA (ng/mL)	17.0 ± 12.3; 14.0: (2.4–66.0)
PSAD	0.28 ± 0.19; 0.20: (0.03–0.48)
Prostate volume (cc)	47.1 ± 20.1; 39.5: (20.0–89.0)
**mMR PIRADS score total cases** PIRADS 2 PIRADS 3 PIRADS 4 PIRADS 5	3 (2.0%) 14 (9.5%) 71 (47.5%) 62 (41.0%)
Prostate Tumor size (mm) at mMR	13.5 ± 5.1; 12.5: (5.4–31.1)
**Clinical T staging** T1c T2 T3a T3b	15 (10.1%) 107 (71.3%) 16 (10.6%) 12 (8.0%)
**Clinical N staging** N0 N1	126 (84.0%) 24 (16.0%)
**Biopsy outcomes** % Positive samples PC % Positive clinically significant PC Max % PC tissue per core	63.7 ± 27.1; 59.8: (9.0–100.0) 56.7 ± 30.5; 52.0: (10.0–100.0) 60.1 ± 28.2; 51.0: (8.0–100.0)
**ISUP grading at biopsy** 1 2 3 4 5	5 (3.3%) 13 (8.7%) 47 (31.7%) 58 (38.0%) 27 (18.3%)
**Nomograms results (% estimated risk for N+)** MSKCC Briganti 2012 Briganti 2017 Briganti 2019	33.5 ± 19.7; 31.0: (5.0–84.0) 26.1 ± 19.7; 19.6: (6.0–85.0) 43.3 ± 25.4; 41.0: (5.0–95.0) 24.9 ± 20.0; 19.0: (5.0–84.0)
**Percentage of patients with estimated risk for N+ at nomogram over the cut-off** MSKCC (>7%) Briganti 2012 (>5%) Briganti 2017 (>7%) Briganti 2019 (>7%)	93.8 % 94.0 % 90.1 % 85.7 %
Number of suspected lymph nodes at imaging	2.8 ± 1.9; 3: (1–6)
**Surgical technique at radical prostatectomy** - Pure Laparoscopic - Robot-assisted	44 (29.0%) 106 (71.0%)
**Pathological stage (T)** pT2 pT3a pT3b pT4	39 (26.2%) 68 (45.1%) 42 (28.0%) 1 (0.7%)
**Pathological stage (N)** N0 N+	111 (73.9%) 39 (26.1%)
**Number of lymph nodes removed at surgery** - Total cases - N+ cases - N0 cases	24.1 ± 9.01; 21: (12–46) 24.3 ± 9.1; 22: (13–45) 23.9 ± 8.9; 23: (11–44)
Percentage of positive lymph nodes	16.1 ± 12.9; 12.0: (5.1-67.3)
**ISUP grading at surgery** 1 2 3 4 5	3 (2.3%) 9 (6.1%) 49 (32.3%) 60 (40.1%) 29 (19.2%)
**Surgical margin at surgery (R)** - Negative - positive	108 (72.0%) 42 (28.0%)
**PNI at surgery** positive negative	78 (52.0%) 72 (48.0%)
**Cribriform/IDC at surgery** - positive - negative	28 (18.7%) 122 (81.3%)
Postoperative total PSA (ng/mL)	0.32 ± 1.45; 0.02: (0.01–5.0)
Biochemical progression	28 (17.3%)
Time to biochemical progression (months)	7.1 ± 10.6; 3.0 (2–25)

Mean ± SD, median, (range). Number of cases (%).

**Table 2:** Comparative analysis based on pN results.

Pathological Lymph Node Status	pN0	pN1	*p* Value
Number cases	111	39	-
Age (years)	65.0 ± 7.1; 67.0: (48–73)	65.5 ± 7.2; 66.0: (50–72)	0.40
BMI	26.3 ± 3.1; 26.9: (22–35)	26.8 ± 3.4; 26.1: (23–37.7)	0.30
Preoperative total PSA (ng/mL)	10.9 ± 10.0; 8.7: (1.6–66.0)	17.6 ± 15.1; 12.0 (3.7–65.2)	0.01
PSAD	0.20 ± 0.7; 0.15: (0.05–0.60)	0.39 ± 0.06; 0.40: (0.35–0.5)	0.06
Prostate volume (cc)	45.6 ± 21.0; 38.0: (23.0–89.0)	48.7 ± 13.5; 49.0: (40.0–65.0)	0.30
**mMR PIRADS score** PIRADS 2 PIRADS 3 PIRADS 4 PIRADS 5	3 (2.0%) 17 (11.2%) 72 (48.3%) 58 (38.5%)	0 (0%) 5 (3.5%) 94 (62.6%) 51 (33.9%)	0.30
Prostate Tumor size (mm) at mMR	14.0 ± 6.2; 12.4:(5.3–31.0)	13.9 ± 6.4; 13.0: (6.9–28.0)	0.50
**Clinical T staging** T1c T2 T3a T3b	17 (11.5%) 116 (77.4%) 10 (6.6%) 7 (4.5%)	0 (0%) 129 (86.1%) 13 (8.5%) 8 (5.4%)	0.004
**Clinical N staging** N0 N1	141 (94.3%) 9 (5.7%)	118 (78.9%) 32 (21.1%)	0.002
**Biopsy outcomes:** % Positive samples PC % Positive clinically significant PC Max % PC tissue per core	59.8 ± 25.8; 58.0: (9.0–100) 54.2 ± 29.5; 50.3: (8.0–100) 56.5 ± 27.6; 50.0: (6.0–100)	62.4 ± 29.8; 62.0: (11.4–100) 52.1 ± 31.5; 49.5: (11.0–100) 71.3 ± 24.1; 67.6: (32.1–100)	0.20 0.30 0.001
**ISUP grading at biopsy** 1 2 3 4 5	5 (3.1%) 18 (12.2%) 47 (31.6%) 57 (37.8%) 23 (15.3%)	4 (2.5%) 20 (13.6%) 47 (31.4%) 55 (36.5%) 24 (16.0%)	0.50
**Nomograms results (% estimated risk for N+)** MSKCC Briganti 2012 Briganti 2017 Briganti 2019	32.1 ± 18.9; 29.0: (4–81) 25.2 ± 19.7; 18.0: (4–80) 42.0 ± 26.6; 39.1: (4–94) 23.5 ± 19.8; 16.9: (4–82)	37.1 ± 18.2; 34.8: (7–75) 28.1 ± 18.5; 21.1: (7–84) 47.8 ± 24.3; 45.9: (7–90) 28.2 ± 21.3; 22.0: (4–78)	0.08 0.20 0.09 0.10
**Percentage of patients with estimated risk for N+ at nomogram over the cut-off** MSKCC (>7%) Briganti 2012 (>5%) Briganti 2017 (>7%) Briganti 2019 (>7%)	92.8% 91.2% 86.4% 80.7%	98.0% 100% 97.5% 97.1%	0.20 0.03 0.04 0.03
Number of suspected lymph nodes at imaging	1.3±0.48; 1.0 (1–2)	2.9±1.6; 3.0 (1–5)	0.03
**Surgical technique at radical prostatectomy** - Pure Laparoscopic - Robot-assisted	23 (15.2%) 51 (33.8%)	21 (13.8%) 55 (36.2%)	0.70
**Pathological stage (T)** pT2 pT3a pT3b pT4	62 (41.2%) 63 (42.3%) 25 (16.5%) 0 (0%)	12 (7.8%) 77 (51.1%) 60 (40.2%) 1 (0.9%)	0.02
Number of Lymph nodes removed at surgery	23.8 ± 8.4; 23.0: (12–46)	25.0 ± 8.1; 24.0: (13–45)	0.20
**ISUP grading at surgery** 1 2 3 4 5	5 (3.2%) 30 (20.2%) 52 (34.5%) 34 (23.0%) 29 (19.1%)	0 (0%) 25 (17.0%) 47 (31.1%) 22 (14.3%) 56 (37.6%)	0.04
**Surgical margin at surgery (R)** - Negative - Positive	113 (75.5%) 37 (24.5%)	90 (60.3%) 60 (39.7%)	0.01
**PNI at surgery** - negative - positive	69 (45.9%) 81 (54.1%)	57 (37.8%) 93 (62.2%)	0.06
**Cribriform/IDC at surgery** - negative - positive	126 (84.3%) 23 (15.7%)	40 (26.8%) 110 (73.2%)	0.02
Postoperative total PSA (ng/mL)	0.14 ± 0.4; 0.02: (0.01–2.8)	0.82 ± 2.9; 0.02: (0.01–4.8)	0.03
Biochemical progression (number of cases and %)	24 (15.8%)	39 (26.1%)	0.05
Time to biochemical progression	10.1 ± 12.8; 7.8: (3–22)	3.2 ± 1.4; 3.1: (3–7)	0.02

Mean ± SD, median, (range). Number of cases (%).

**Table 3:** Pearson correlation coefficient among pathological N stage (pN) and the other clinical and pathological variables.

Correlation	Coefficient	*p* Value
pN-age	−0.0353	0.670
pN-BMI	0.0524	0.524
pN-prostate volume	0.1577	0.545
pN-risk class	0.0511	0.534
pN-preoperative PSA	0.2155	0.008
pN-PSAD	0.4878	0.055
pN-PIRADS score	0.1275	0.215
pN-prostate tumor volume at imaging	0.0064	0.950
pN-percentage positive core at biopsy	0.0358	0.663
pN-MSKCC nomogram	0.119	0.149
pN-Briganti 2012 nomogram	0.0762	0.390
pN-Briganti 2017 nomogram	0.1188	0.192
pN-Briganti 2019 nomogram	0.1175	0.251
pN-number of suspected N at imaging	0.3313	<0.001
pN-surgical technique	0.1979	0.0152
pN-pT stage	0.3148	<0.001
pN-ISUP grading at surgery	0.1622	0.049
pN-number of lymph nodes removed at surgery	0.0793	0.334
pN-surgical margins	0.2887	0.00034
pN-PNI	0.1249	0.127
pN-cribriform/IDC	0.143	0.08
pN-postoperative PSA	0.2068	0.013

**Table 4:** Sensitivity, specificity, Positive predictive value (PPV), Negative predictive value (NPV), and AUC of different variables in predicting pN+ status at surgery.

	Sensitivity (CI 95%)	Specificity (CI 95%)	PPV (CI 95%)	NPV (CI 95%)	AUC (CI 95%)
**MSKCC nomogram > 7%**	0.973 (0.845–1.000)	0.078 (0.043–0.147)	0.248 (0.181–0.340)	0.905 (0.851–0.938)	0.526 (0.489–0.562)
**Briganti 2012 nomogram > 5%**	0.991 (0.889–1.000)	0.093 (0.049–0.171)	0.285 (0.220–0.351)	0.957 (0.911–0.991)	0.548 (0.518–0.578)
**Briganti 2017 nomogram > 7%**	0.973 (0.840–1.000)	0.140 (0.090–0.230)	0.352 (0.251–0.487)	0.919 (0.851–0.959)	0.555 (0.509–0.601)
**Briganti 2019 Nomogram > 7%**	0.959 (0.789–1.000)	0.183 (0.124–0.291)	0.301 (0.212–0.408)	0.931 (0.855–0.972)	0.573 (0.513–0.633)

**Table 5:** Logistic regression analysis to identify predictors for positive lymph node (pN+).

		Univariable				Multivariable			
		OR	95% CI_lower	95% CI_upper	*p*-Value	OR	95% CI_lower	95% CI_upper	*p*-Value
**Preoperative PSA**		Ref	_	_	_				
	>10	1.771	0.818	3.807	0.141				
**MSKCC**		Ref	_	_	_				
	>7%	3.000	0.371	23.561	0.401				
**Briganti 2012**		Ref	_	_	_				
	>5%	7.752	0.359	159.823	0.210				
**Briganti 2017**		Ref	_	_	_				
	>7%	5.610	0.611	42.812	0.213				
**Briganti 2019**		Ref	_	_	_				
	>7%	5.565	0.786	45.235	0.135				
**Pathologic stage**	pT2	Ref	_	_	_	Ref	_	_	_
	pT3a	6.724	1.932	24.485	0.003	6.52	1.825	24.001	0.005
	pT3b	14.129	3.651	54.231	0.001	11.211	2.621	42.308	0.002
**ISUP at surgery**	1	Ref	_	_	_				
	2	3.845	0.161	96.270	0.502				
	3	4.215	0.1812	98.528	0.413				
	4	2.759	0.111	71.521	0.499				
	5	7.775	0.357	186.632	0.310				

Odds Ratio (OR), 95% Confidential Interval (CI).

**Table 6:** Studies investigating the accuracy of most frequently used nomograms to predict the probability of lymph node metastases before radical prostatectomy.

Study	Sensitivity	Specificity	PPV	NPV	Accuracy AUC (CI 95% Range)
**Hinev et al.** **2014** [11]	N.R.	N.R.	N.R.	N.R.	**Briganti 2012:** 87.5 **MSKCC:** 77
**Bandini et al.** **2017** [12]	**Briganti 2012:** 90.0 **MSKCC:** 89.9	**Briganti 2012:** 46.1 **MSKCC:** 46.4	N.R.	**Briganti 2012:** 98.7 **MSKCC:** 98.7	**Briganti 2012:** 79.8 **MSKCC:** 79.9
**Hueting et al.** **2018** [13]	N.R.	N.R.	N.R.	N.R.	**Briganti 2012:** 76 (73–79) **MSKCC:** 75 (72–78)
**Gandaglia et al.** **2020** [18]	N.R.	N.R.	N.R.	N.R.	**Briganti 2019:** 79 **Briganti 2017:** 75 **Briganti 2012:** 65 **MSKCC:** 74
**Diamand et al.** **2020** [14]	N.R.	N.R.	N.R.	N.R.	**Briganti 2019:** 80 (75–86) **Briganti 2012:** 80 (74–87)
**Milonas et al.** **2020** [8]	**MSKCC:** 88.9	**MSKCC:** 45.2	**MSKCC:** N.R.	**MSKCC:** 96.8	**MSKCC:** 79 (73.8–84.2)
**Oderda et al.** **2020** [15]	N.R.	N.R.	N.R.	N.R.	**Briganti 2019:** 76 (70–81) **Briganti 2017:** 80 (75–86) **Briganti 2012:** 83 (81–84) **MSKCC:** 83 (81–84)
**Fukagawa et al.** **2021** [19]	**Briganti 2019:** 94.7	**Briganti 2019:** 32.0	N.R.	**Briganti 2019:** 98.8	**Briganti 2019:** 71 **Briganti 2017:** 72 **Briganti 2012:** 74 **MSKCC:** 73
**Meijer et al.** **2021** [16]	N.R.	N.R.	N.R.	N.R.	**Briganti 2019:** 82 (76–87) **Briganti 2017:** 76 (70–82) **MSKCC:** 77 (72–83)
**Frego et al.** **2022** [20]	N.R.	N.R.	N.R.	N.R.	**Briganti 2019:** 82 **Briganti 2012:** 84

PPV = positive predicted value; NPV = negative predicted value; AUC = area under the curve; N.R. = not reported.

## Data Availability

The data presented in this study are available on reasonable request from the corresponding author.

## Abbreviations

PC = prostate cancer; RP= radical prostatectomy; LNI = lymph node invasion; LNs = lymph nodes; SM = surgical margins; ePLND = extended pelvic lymph node dissection; mpMRI = multiparametric magnetic resonance imaging; RARP = robot-assisted radical prostatectomy (RARP); LRP = laparoscopic radical prostatectomy; ROC = receiver operating characteristic; AUC = area under the curve; PPV = positive predictive value; NPV = negative predictive value.
